# Supplementary material for: New AI-algorithms on smartphones to detect skin cancer in a clinical setting—A validation study
Source: PLoS One. 2023 Feb 15;18(2):e0280670. doi: 10.1371/journal.pone.0280670 (PMC9931135; doi:10.1371/journal.pone.0280670)
Supplement: S1 File — (DOCX) [file pone.0280670.s001.docx]

Supplementary Material

## 1. Algorithm, model, modelling, training and evaluation

**1.1 Model architecture**

The *analyze* network is an image classifier based on an inception_v3 model. The image classification assesses the whole image and accepts images of single skin lesions as input. Images are therefore decoded and resized to 299 (x-axis) x 299 (y-axis) x 3 (RGB) pixels to fit the input requirements of the inception model. Based on the input, the algorithm returns probability scores for each of the possible 47 lesions. Each score lies between 0% and 100% and reflects the probability of being the correct diagnosis. All scores of a lesion add up to 100%. The assessment is performed in significantly less than a second and can also be used in real-time like for video input.

The *detect* network is based on the faster_rcnn_inception_v2_coco model. It accepts images containing one or several skin lesions as input and returns a list of bounding boxes, a label assigned to each box and a corresponding probability. It consists of three components: convolution layer, region proposal network (RPN), and “classes and bounding boxes prediction”. The convolution layer serves as an initial filter for relevant features of the image. The RPN is a small network, that localizes objects in the image and generates boxes around these objects. The “classes and bounding boxes prediction” calculates the label probabilities for each box and returns the most likely diagnosis, together with its probability. Finally, overlapping boxes are removed. The decision, which box to keep, is based on 1) higher risk and 2) higher probability score. The cumulative probability of all labels can exceed 100% as each box is evaluated separately.

**1.2 Data sources**

An image data set of 19,576 anonymized images, was used. It was split (the training script splits the data by random) into a training and validation data set (18,384 dermatologists labeled images and a test data set (n=1,192) in order to ensure that test data set images are not used in both training and validation of the model the filenames of the test data set images start with tds*.jpg.
The image data set (n=19,576) contains anonymized representative (age of participants: 18 to 86 years, Fitzpatrick skin type 1 to 4, a clear contrast to surrounding skin, not covered by hair or opaque/glittering substances, not previously traumatized except for label „skin injury") images.
The test data set (n=1,192), which was not used for training, contains anonymized representative images (age of participants: 18 to 86 years, Fitzpatrick skin type 1 to 4, a clear contrast to surrounding skin, not covered by hair or opaque/glittering substances, not previously traumatized).

**1.3 Model training**

The models (both, analyze and detect) were retrained using the training data set for CENSORED iterations. The training process runs through the training image data set in batches and iterations, with validation run on each iteration.

The following settings were used for analyze network to train:

- learning_rate: CENSORED
- testing_percentage: 10
- validation_percentage: 10
- eval_step_interval: 10
- train_batch_size: CENSORED
- test_batch_size: CENSORED
- validation_batch_size: 100

The following settings were used to train the detect network:

- truncated_normal_initializer stddev: 0.01
- first_stage_nms_score_threshold: 0.0
- first_stage_nms_iou_threshold: 0.7
- first_stage_max_proposals: 300
- first_stage_localization_loss_weight: 2.0
- first_stage_objectness_loss_weight: 1.0
- initial_crop_size: 14
- maxpool_kernel_size: 2
- maxpool_stride: 2
- dropout: false
- dropout_keep_probability: 1.0
- fc_hyperparams l2_regularizer weight: 0.0
- variance_scaling_initializer factor: 1.0 uniform: true mode: FAN_AVG
- second_stage_post_processing batch_non_max_suppression score_threshold: 0.0
- iou_threshold: 0.6
- max_detections_per_class: 100
- max_total_detections: 300
- score_converter: SOFTMAX
- second_stage_localization_loss_weight: 2.0
- second_stage_classification_loss_weight: 1.0
- batch_size: CENSORED
- optimizer momentum_optimizer
- learning_rate: manual_step_learning_rate
- initial_learning_rate: CENSORED
- momentum_optimizer_value: 0.9
- use_moving_average: false
- gradient_clipping_by_norm: 10.0
- eval_config: num_examples: 381
- max_evals: 10

**1.4 Model evaluation**

The models were tested with test dataset (n=1,192) to measure sensitivity and specificity for detecting the risk classes. None of the images in the test dataset was used to train the model.

| network type | sensitivity | specificity |
| --- | --- | --- |
| ANALYZE | 91,2% | 94,9% |
| DETECT | 93,8% | 96,8% |

2. Case number planning

Assumptions for case number planning:

We assume 85% sensitivity for melanoma and squamous cell carcinoma, respectively, and 70% sensitivity for basal cell carcinoma.

No possible drop-outs are considered in case planning.

3. Sample size planning

With a sample size of 196, the sensitivity of 85% (70%) can be estimated with an accuracy of 5% (6.4%).

With a sample size of 323, the sensitivity of 85% (70%) can be estimated with an accuracy of 3.9% (5%).

Further, it should be considered whether to stratify by different skin types.

For the case number calculation, nQuery Advisor + nTerim 4.0 was used (two-sided 95% confidence interval for a proportion with normal distribution approximation).

4. Calculation “High and medium risk versus low risk”

a) Sensitivity:

"Analyze" mean: 97.9% (CI 96,.7-99.2)

"Detect" mean: 96.1% (CI 93.6-98.6)

b) Specificity:

"Analyze" mean: 98.6% (CI 97.6-99.6)

"Detect" mean: 97.7% (CI 95.9-99.5)
