# Supplementary material for: New AI-algorithms on smartphones to detect skin cancer in a clinical setting—A validation study
Source: PLoS One. 2023 Feb 15;18(2):e0280670. doi: 10.1371/journal.pone.0280670 (PMC9931135; doi:10.1371/journal.pone.0280670)
Supplement: Attachment — Submitted filename: Reply letter.docx [file pone.0280670.s003.docx]

Teresa Maria Kränke, MD

Department of Dermatology and Venereology

Medical University of Graz, Austria

E-mail: [teresa.kraenke@medunigraz.at](mailto:teresa.kraenke@medunigraz.at)

Phone: +43 316 385 12423

Fax number: +43 316 385 14822

Graz, 14^th^ June 2022

To the Editor

RE: Submission of the revised version of the manuscript number **PONE-D-22-08100**

Dear Editor,

Thank you for giving us the opportunity to re-submit our manuscript titled **“New AI-algorithms on smartphones to detect skin cancer in a clinical setting”.** Please find below the point-to-point answers to the suggestions made by the reviewers.

Reviewer #1:

This paper describes an evaluation of two AI algorithms for skin cancer diagnosis. Unfortunately, it is not well reported and does not meet available reporting guideline for test accuracy studies. I recommend that the study (and abstract) is at least reported in accordance with the STARD reporting guideline. Unfortunately, the extension to cover AI tools is not yet published, however the authors could also consider looking at the MI-CLAIM checklist for guidance on what is needed for reporting of an AI tool (https://doi.org/10.1038/s41591-020-1041-y). It is actually quite difficult to tell whether the authors consider the paper tor report both development and validation of the AI tools or validation only – there is a paucity of detail in regard to several aspects of the study making it really quite difficult to review. It seems possible that plans to commercialise the AI algorithms may have led to details being withheld from the report. Given the potential impact of using this type of algorithm on patients I would encourage authors to report sufficient details of both the development and validation of the tools to allow the methods and results to be fully judged and to support any subsequent utilisation of these tools.

**Authors response: We thank the reviewer for these comments. We have not done a developing/evaluation study, but rather a study on the clinical performance of two CE-certified algorithms using artificial intelligence. In their developing phase the algorithms were trained on 18.384 images labeled with one of 47 distinct subcategories in order to accurately distinguish benign and malignant skin lesions. Please refer to the supplement for further information and to the lines 35-49.**

**We have referred to the STARD guidelines wherever we considered useful.**

**Furthermore, we have provided details about function principles of both models in the supplementary material. As both algorithms are already CE-certified, the subject of our study was the validation / clinical performance of both modes; we therefore did not provide more information about the development or function principles.**

Title – should reflect that this is diagnostic test accuracy study, please reword
Abstract – please refer to the STARD extension for Abstracts. Taking this as a report of a validation study only, then missing items include details about eligibility criteria for participants and lesions, setting (ie it is a dermatology referral centre), participant recruitment method, and description of the index test and reference standard. The Results should report the number of ‘cases’ amongst the evaluated lesions, and neither Results nor Conclusions adequately report that the AI tools are intended to assess the risk of a skin lesion being malignant.

**Authors response: We thank the reviewer for these comments.**

**Title: We reworded it according to your suggestion. Abstract: Our study is a proof of concept of AI-algorithms already fulfilling the CE-criteria and is registered as medical product at the Austrian Federal Office for Safety in Health Care (registration number: 10965455). Consequently, we did not perform an evaluation study in the narrower sense, but rather a validation study/testing of the performance of already certified AI algorithms in a clinical setting. However, we referred to the STARD guidelines wherever we considered useful. Concerning your request on a more detailed description of participants´ recruitment and lesions´ eligibility criteria, we have added the missing information. In order not to exceed the abstract, we have made added the number of “cases” in the main manuscript and made a table. We reworded the conclusion according to your suggestions.**

Introduction
Line 2 – I believe that ‘keratinocyte’ skin cancer is now the more generally accepted term for ‘non-melanoma’
Line 8 – Could add caveat that BCC and SCC have a ‘generally’ favourable prognosis or similar
Line 25 – The Rezazade Mehrizi reference (15) is not the most appropriate reference to support the statement about potential value of AI tools across sectors
Line 35-40 – This paragraph about programming of a CNN and novel stratification CNN implies that the development phase for the AI tools evaluated in this study has already been conducted, is that correct? Very limited detail is provided about the development, training and validation of the two models either here or in the rest of the paper or supplement. Reference 28 (Han 2018) cited in this paragraph does not have any authors in common with this paper so presumably reports a similar method rather than the development of either model used here. It is essential that the development of AI tools is transparently reported so that readers can be satisfied that such tools have been rigorously and robustly evaluated (this equally applies to tools intended for commercial use).

**Authors response: We thank the reviewer for these comments.**

**Line 2: In Europe the term „non-melanoma skin cancer“ is well established as it not only includes tumors of keratinocyte origin (e.g., squamous cell carcinomas), but also**

**others like Merkel cell carcinomas or basal cell carcinomas. Following this, we didn´t change this phrase as suggested by you.**

**Line 8: We have changed this sentence accordingly.**

**Line 25: We have added an additional reference to adequately support the statement about AI as helpful tool. See new reference number 16 (Tomas et al.) (see line 15/16).**

**Line 35-40: The tested AI-algorithms are in line with the CE-criteria and registered as medical product at the Austrian Federal Office for Safety in Health Care (registration number: 10965455). We performed the first evaluation study of these algorithms in a clinical setting with “high-risk” patients at one tertiary referral center; hence, the intention of our study was to evaluate the performance of these algorithms in a clinical setting. Considering this, we did not provide many data about the development and training of the two models. The method of reference 28 was modified for our study purposes and was not topic of this paper.**

Methods
A number of standard elements for any DTA evaluation are missing from this section. I have highlighted a few but suggest the authors refer to the STARD reporting guideline for further information about this.
Line 50-51 – State that histology or clinical diagnosis was the reference standard; more details needed in regard to selection for histology – was this independent of the result of the two index tests or not? If so, how was this implemented? Discussion also mentioned follow up of dysplastic nevi, why was is this not mentioned in the methods?
Line 57-58 – There is potentially quite a difference between participants scheduled for preventive skin examination (who may have no suspicious/biopsied skin lesions) and those who were scheduled for removal of at least one skin lesion. Please explain more about how participants were included/excluded and whether recruitment was consecutive or not. For the former group in particular, did all participants contribute at least one suspicious lesion? How many ‘benign appearing’ lesions were sampled per patient? It is very important to understand the potential for selection bias and the spectrum of included participants in order to judge the applicability of results.
Line 62-69 – This section describes both the reference standard and acquisition of images for the index tests – these should be separate processes that are described separately and in more detail.
Line 62-64 More information is needed about how the clinical diagnoses were made and the result recorded (did the same two dermatologists see all patients?) as this forms part of the reference standard. How were lesions selected for biopsy? How was the reference standard result categorised as ‘diseased’ versus ‘not diseased’, did diseased include clinical diagnosed BCCs for example, or were only histologically confirmed malignancies considered as disease positive.

Line 64-69. How were lesions selected for imaging? How did clinicians decide which of the several smartphones to use for imaging? Did the same two clinicians carry out the imaging as those who made the clinical diagnoses. Lack of blinding between index and reference standard and between the two index tests is potentially a major source of bias here.

Line 71-80 and Supplement – Detail of algorithm development is too meagre to really allow any comment to be made.
Line 81 Statistical analysis – What sample size considerations were made for the study?
Line 92-93 What is the justification for excluding lesions rated as ‘benign’ from specificity calculations?

**Authors response: We thank the reviewer for these comments.**

**We referred to the STARD guidelines wherever we considered useful.**

**Line 50-51: Histology was the reference standard; we have added this point. If both dermatologists evaluated a lesion as being benign, no histology was performed; consequently, the last decision (whether to biopsy/excise a lesion or not) was always made by the two dermatologists, even if the algorithm made an opposite risk classification. The follow-up of non-excised dysplastic nevi was added to this paragraph (see lines 59-68).**

**Line 57-58: We partially agree with you in that point; however, as we are a tertiary referral center, we have already “pre-selected” patients; meaning that we usually see patients at high risk for developing skin cancer of any type (e.g., patients with multiple and/or atypical nevi or patients with severe sun-damaged skin). Considering this, our study population was not that heterogeneous. We have adapted the paragraph (see lines 73-75).**

**No, not every patient from the group “scheduled for preventive skin examination” had a suspicious lesion to be biopsied/excised. Five lesions (both, benign and malignant) were scanned per patient averagely – we have already mentioned that in the results section and additionally added this information to this paragraph (see line 76).**

**Line 62-69: We have provided this information in the supplement.**

**Line 62-64: The two dermatologists did a state-of-the-art clinical/dermatological examination screening the entire skin, the palms and soles and the scalp. However, this clinical examination was not always done by the same two dermatologists. As the clinical examination was performed by experienced dermatologists, they selected the lesions for biopsy based on distinct clinical/dermoscopic malignancy-criteria. As we have mentioned above, the last decision was always made by the clinicians regardless of the algorithm’s risk classification. The histology was always considered as reference standard, however, a small proportion of patients denied biopsy/excision of a suspicious lesions. In these cases, the diagnosis of the two dermatologists was considered as reference; we have added this point (see lines 61-66).**

**Line 64-69: The lesions for imaging were randomly selected by the evaluating dermatologists. Notably, no one, who did the index testing has screened the patients.**

**Line 71-80: As already mentioned, we performed an evaluation study of an already registered medical product fulfilling CE-criteria; further information concerning model training, evaluation and testing is given in the completed supplementary.**

**Line 81: The sample size planning was performed by the Institute for medical statistics at the Medical University of Graz; we have added this information to the supplementary.**

**Line 92-93: We excluded the lesions rated as “benign” in one calculation in order to differentiate the groups “medium / yellow” and “high / red” adequately. We added this point (see lines 109-111).**

Results
No information is provided about the participants other than age group – additional demographic and clinical characteristics are potentially very important for skin cancer diagnosis.
Importantly the final lesion diagnoses were not reported so the reader has no idea how many lesions had a final ‘malignant’ diagnosis nor what type of lesions the authors included in this definition. From the 95% CIs reported, over 300 malignant lesions must have been included in the sample which does imply a highly selected sample.
No information is given about how successful the imaging was (how many attempts needed to get a good image), nor any failure rate for the AI tools. According to the used mobiles with a very good camera

No absolute numbers to support the results are provided, only accuracy, sensitivity and specificity – this needs to be much more detailed to allow results to be checked and to provide more informative results.
Discussion – In my opinion, without the further detail requested in regard to the study methods and results it is not really possible to comment on the Discussion and author conclusions however it does appear that the significance of the results have been rather over-stated.

**Authors response:**

**We thank the reviewer for these comments. Besides age groups, we have already provided data about the gender distribution. We additionally added the distribution of skin types throughout the study population (see lines 120-123). More information was not gathered per patient as this was not the primary subject of our study; moreover, the algorithms only evaluated the lesions without any patients´ information.**

**We have provided the detailed allocation of the lesions to the respective risk categories including the absolute numbers (see table 2); we have moreover added the types of lesions in the “medium” and “high” risk group. Concerning your objection on a highly selected sample: Our study was performed in a clinical setting with primary aim to detect malignant lesions with a high sensitivity. We have therefore chosen this setting knowing that this is not fully applicable for the “general population”. We agree with you that this is a further limitation and have added this point in the limitations-section (see lines 202-205).**

**The imaging procedure was easily done in most of the cases and usually no more than one attempt per lesion was needed in order to get a good image without any failure. The average imaging time including the recording of patient´s data was three minutes. We have added this information (see lines 134-137).**

Reviewer #2:

The article is interesting, innovative and relevant, but some important information to analyze the data is missing.
I would suggest you make a Table with the frequency of images in each of the five categories and how many of them had the clinical evaluation or histopathological report. Also, it was not clear if you considered in your results all of the 238 patients or only those with biopsy or clinical evaluation?
Another question: did the dermatologists classified the images in low, medium and high risk or they were assigned automatically according to the diagnosis?
The agreement rate of diagnostic and risk classification could be stratified according to the lesions that were seen by dermatologists and had the histopathological report too. It is critical to know how the ground truth is being assigned to compare with the algorithm´s accuracy.
Reference 20 is equal to 22.

**Authors response: We thank the reviewer for these comments. We have created a table based on your suggestions. All of the included 238 patients (irrespectively of having had a biopsy/excision) were considered for statistical analysis.**

**Yes, the dermatologists made a diagnosis and afterwards also classified the lesions in low, medium and high.**

**We have deleted one reference and adapted the others accordingly.**

**Editors’ comments:**

When submitting your revision, we need you to address these additional requirements.

1. Please ensure that your manuscript meets PLOS ONE's style requirements, including those for file naming. The PLOS ONE style templates can be found at 
<https://journals.plos.org/plosone/s/file?id=wjVg/PLOSOne_formatting_sample_main_body.pdf> and https://journals.plos.org/plosone/s/file?id=ba62/PLOSOne_formatting_sample_title_authors_affiliations.pdf

2. Thank you for stating the following in the Competing Interests section:

"Michael Koppitz and Michael Tripolt share a company founded after finishing the study to produce a consumer-usable early skin cancer detection app. The remaining authors have no conflicts of interest to declare. "

Please confirm that this does not alter your adherence to all PLOS ONE policies on sharing data and materials, by including the following statement: "This does not alter our adherence to  PLOS ONE policies on sharing data and materials.” (as detailed online in our guide for authors http://journals.plos.org/plosone/s/competing-interests).  If there are restrictions on sharing of data and/or materials, please state these. Please note that we cannot proceed with consideration of your article until this information has been declared.

Please include your updated Competing Interests statement in your cover letter; we will change the online submission form on your behalf.

Additional Editor Comments:
In accordance with the expert reviewers, I have a number of both technical and conceptual concerns. Rather than repeat those points here, I refer you to the specific remarks (below) for details.

**Authors response: We thank the Editor for these comments.**

**1.** We have revised our manuscript in order to meet the PLOS ONE's style requirements.

**2.** We have included the abovementioned Competing Interests statement to our updated cover letter.

**3.** We have addressed all your technical and conceptual concerns as also mentioned by the reviewers.

Thank you for your help and re-consideration

Sincerely yours,

Teresa Kränke, MD (corresponding author)

Michael Tripolt, MD, MPH

**Conflicts of interest:** Michael Koppitz and Michael Tripolt share a company founded after finishing the study to produce a consumer-usable early skin cancer detection app. This does not alter our adherence to PLOS ONE policies on sharing data and materials. The remaining authors have no conflicts of interest to declare.

**Funding source:** The development of the algorithms was funded by the amiflow Ltd., Graz, Austria; however, the authors did not receive any salaries by this company nor did it have any influence on the study design, data collection and analysis, decision to publish, or preparation of the manuscript.
