# Supplementary material for: New AI-algorithms on smartphones to detect skin cancer in a clinical setting—A validation study
Source: PLoS One. 2023 Feb 15;18(2):e0280670. doi: 10.1371/journal.pone.0280670 (PMC9931135; doi:10.1371/journal.pone.0280670)
Supplement: Attachment — Submitted filename: Response to Reviewers.docx [file pone.0280670.s004.docx]

Teresa Maria Kränke, MD

Department of Dermatology and Venereology

Medical University of Graz, Austria

E-mail: [teresa.kraenke@medunigraz.at](mailto:teresa.kraenke@medunigraz.at)

Phone: +43 316 385 12423

Fax number: +43 316 385 14822

Graz, 18^th^ September 2022

To the Editor

RE: Submission of the revised version of the manuscript number **PONE-D-22-08100R1**

Dear Editor,

Thank you for giving us again the opportunity to re-submit our manuscript titled **“New AI-algorithms on smartphones to detect skin cancer in a clinical setting”.** Please find below the point-to-point answers to the suggestions made by the reviewers.

Reviewer #1:

Thank you for the opportunity to review this revised submission. Although some changes have been made, I remain confused about several aspects of the study which I have documented below. One of the most important omissions is lack of data regarding lesion types, how the reference standard was reached for each group, and lack of data underlying the main accuracy results making it impossible to check the authors claims.
1. Additional information is provided about the two algorithms used, namely that they were previously developed and internally validated by the study authors and both are CE certified. The algorithms are identified as ‘analyze’ and ‘detect’ and seem to be incorporated into a smartphone app for skin cancer detection/risk assessment but the app itself is not identified. The supplementary information provides some information about the development and validation process however full details are not provided and this information does not appear to be in the public domain. As per my comment on the previous iteration of this paper, ideally one would one to see sufficient details of both the development and validation of the tools to allow the methods and results to be fully judged and to support any subsequent utilization of these tools. As it stands it is not possible to associate these algorithms with any CE marked software or app.

**Author response: We thank the reviewer for these comments. Unfortunately, we are restricted to publicly share more data concerning the development and validation process of these both algorithms as this would infringe the copyright of both, the** **amiflow Ltd., Graz (funded the development) and the medaia Ltd., Graz (developer and owner of the App).
Moreover, as mentioned in the paper and the previous reply letter, we only performed a validation study of an already developed and CE-conform App.**

**The developed App (Skinscreener©), however, is publicly available in the Google Play Store and the Apple Store. Please refer to: https://skinscreener.com/**

2. Additional information is provided about methods for reaching a reference standard diagnosis for the included lesions, however the Results section does not report actual final diagnoses nor how they were reached (histology, clinical dx by two dermatologists or follow-up). It is also notable that the authors state that histology was the reference standard however only 165 lesions were reported as having a histological diagnosis. This is an important omission that needs to be addressed. It also appears that the clinical diagnosis was made in knowledge of the of algorithm recommendation (the last decision … was always made by the two dermatologists, even if the algorithm made an opposite risk classification). The lack of blinding to the algorithm recommendation must be clearly stated in the study report as, even with the best will in the world, knowledge of the index test result has clear potential to influence clinical decision making. The beginning of section ‘Procedure and Mobile Devices’ implies that clinical decision may have been made prior to use of the algorithm and could therefore have ensured some level of blinding – if this was the case then it should be stated more clearly.

**Author response: We thank the reviewer for these comments. Concerning the final diagnoses of the lesions, we have added another table (table 3).**

**We stated the histology as ideal endpoint; in detail: Only those lesions, which were evaluated as being suspicious by the dermatologists, were removed – this procedure explains the low number of histologically proven lesions compared with the number of included lesions. We have adapted that paragraph (see lines 60-64).**

**All lesions were first evaluated by two dermatologists and afterwards by the algorithms operated by a third dermatologist. Your concerns about the blinding and influence of the clinical decision are completely understandable, however, as the evaluating dermatologists had no idea about the algorithms´ evaluation the criteria for an adequately blinding-process are fulfilled in our view. According to your suggestion we have clarified the paragraph in the section “Procedure and Mobile Devices” (see lines 86-90).**

3. Helpful clarification about the nature of the study population was provided. The recruitment process was still not described however (e.g., consecutive, convenience etc), and as per comment above, the actual final diagnoses of the included lesions were not reported.

**Author response: We thank the reviewer for these comments. The recruitment process was consecutive; in detail: Every patient, who was seen by one of the study authors in the mentioned time period (June 2018-December 2019) was ask to participate in this study. We added this information (see lines 72-75).**
**Concerning the final diagnoses please refer to table 3.**

4. I do not understand the authors’ meaning in the following sentence in their response (re lines 64-69) “Notably, no one, who did the index testing has screened the patients.”
**Author response: We thank the reviewer for this comment. This sentence indicates, that no author, who screened the respective patient did the screening with the smartphone. Please also refer to point 2 in this reply letter.**

5. I would still like to see a clearer separation of the description of the algorithms and their use from the reference standard diagnoses. I think that the algorithms provide both a diagnosis of lesion type and a risk classification but this has to be inferred from a number of different sections (line 58-59, 81-85, 87-96, 125-129) and could be more clearly documented.

**Author response: We thank the reviewer for this comment. As you mentioned, these phrases were not clearly worded. The algorithms only provide a risk classification (benign/green, medium/yellow, high/red) of a respective lesion. In a previous training phase, both were trained with 18.384 lesions, which were allocated to one of the above-mentioned risk categories in each case. A list of lesions assigned to the risk groups “high” and “medium” is already given in the section “patients´ and lesions´ characteristics”. The lines you mentioned were reworded, wherever we considered useful.**

6. The authors’ justification for excluding lesions rated as “benign” from calculation of specificity is inadequate. The paper also states that specificity was calculated both including and then excluding images of the risk category “benign response” but I cannot find this in the paper. An alternative approach would be to have two definitions of ‘index test positive’ for calculation of both sensitivity and specificity, i.e. high risk (index positive) versus medium/low risk (index negative), and high or medium risk (index positive) versus low risk (index negative).

**Author response: We thank the reviewer for this comment.**

**The results concerning the specificity (both, including and excluding the lesions of the risk category “benign” are mentioned in the section “Results/accuracy of the algorithms” (see lines 163-166).**

**We agree with you that your approach would also be feasible; however, we mainly did the calculation as reported for the following reasons: In clinical practice the main goal is to identify possibly malignant, meaning suspicious lesions, as they require further medical action and/or treatment. Following this, it does not matter whether the suspicious lesions is allocated to the “yellow/medium” or the “red/high” risk group, as the respective lesion in any case has to be treated (i.e., follow-up in case of atypical nevi or histological examination in basal cell carcinoma). On the other hand, there is no need for action regarding lesions assigned to the “green/low” risk group.**

**In our view, our approach corresponds in principle to your suggestion to calculate high or medium risk (index positive) versus low risk (index negative). However, as you suggested, we have calculated both, the sensitivity and specificity for “high and medium risk versus low risk” and added this information to the supplementary material.**

7. Table 2 provides the number of lesions per risk category as determined by each algorithm, and the number of lesions with a histological diagnosis, however these are the only absolute numbers provided. Ideally one would want to see a tabulation of the 47 lesion subcategories (or at least the five categories) against algorithm classification, i.e., number of lesions categorized as high/medium/low by the algorithm per lesion category. In addition, the absolute numbers underlying each percentage should be given e.g., for sensitivity 95.35%, the number of lesions classed as true positive and the total number of ‘malignant’ lesions should be reported. This is a very important omission that must be addressed. It should also be made crystal clear that the accuracy data reported is for correct diagnosis of ‘possible malignancy’ as opposed to detection of already malignant skin lesions (i.e., includes detection of actinic keratosis or dysplastic nevus which are not universally considered even as ‘pre-malignant’).

**Author response: We thank the reviewer for these comments. Based on your suggestions we have added another table (table 3) and you will find there all missing information.**

8. Related to the above point, the Discussion mentions results that are not presented in the Results sections as far as I can see (i.e., lower ‘accuracy’ for detection of malignant lesions).

**Author response: We thank the reviewer for this comment and totally agree with you that some data presented in the discussion were missing in the results. We have added that (see lines 166-168).**

Reviewer #2: I found that the manuscript has gotten much better now after the review, but I still miss more data in the Results Section. I believe it is important to have a Table showing how many lesions were classified as benign and non-benign, including how many BCCs, SCCs, Melanomas, and how many of those had proven histopathological exam or only clinical diagnosis.

**Author response: We thank the reviewer for these comments. According to your suggestion we have added another table (table 3) containing all the missing information.**

Additional Editor Comments:
I thank the authors for their revision. I agreement with the expert reviewers, there are still a number of concerns that need to be addressed. Please refer to the reviewers’ comments below.

**Author response: We thank the Editor for these comments. We have addressed all concerns made by the reviewers.**

**We have created table 3 containing all missing information mentioned by the reviewers and added it to the main manuscript; however, this table could also be added to the supplementary material – whatever the Editor likes better.**

Thank you for your help and re-consideration

Sincerely yours,

Teresa Kränke, MD (corresponding author)

Michael Tripolt, MD, MPH

**Conflicts of interest:** Michael Koppitz and Michael Tripolt share a company founded after finishing the study to produce a consumer-usable early skin cancer detection app. This does not alter our adherence to PLOS ONE policies on sharing data and materials. The remaining authors have no conflicts of interest to declare.

**Funding source:** The development of the algorithms was funded by the amiflow Ltd., Graz, Austria; however, the authors did not receive any salaries by this company nor did it have any influence on the study design, data collection and analysis, decision to publish, or preparation of the manuscript.
