# Supplementary material for: New AI-algorithms on smartphones to detect skin cancer in a clinical setting—A validation study
Source: PLoS One. 2023 Feb 15;18(2):e0280670. doi: 10.1371/journal.pone.0280670 (PMC9931135; doi:10.1371/journal.pone.0280670)
Supplement: Attachment — Submitted filename: Response to Reviewers.docx [file pone.0280670.s005.docx]

Teresa Maria Kränke, MD

Department of Dermatology and Venereology

Medical University of Graz, Austria

E-mail: [teresa.kraenke@medunigraz.at](mailto:teresa.kraenke@medunigraz.at)

Phone: +43 316 385 12423

Fax number: +43 316 385 14822

Graz, 23^th^ December 2022

To the Editor

RE: Submission of the revised version of the manuscript number **PONE-D-22-08100R2**

Dear Editor,

Thank you for giving us again the opportunity to re-submit our manuscript titled **“New AI-algorithms on smartphones to detect skin cancer in a clinical setting”.** Please find below the point-to-point answers to the suggestions made by the reviewers.

Reviewer #1:

Thank you for the opportunity to review this revised submission. The paper is greatly improved and most of my earlier comments now addressed. The additional information provided regarding blinding, application of the reference standard and participant recruitment is very helpful.

1. I understand and accept that in this situation it is not possible to report information regarding earlier development and validation of the algorithms evaluated in this study, however it does not seem unreasonable to expect the authors to identify the algorithms’ association with Skinscreener app in the text of the paper, or is this also not possible?
**Author response: We thank the reviewer for this comment. The used AI in our study is the core of the App Skinscreener©, but not the App itself – in other words, the AI is the motor of the App, therefore we think it is not reasonable to identify the algorithms´ association with the App. Moreover, with the end of this year an updated version of the App will be available in the Google Play Store and the Apple Store.**

2. Table 3 is an important and very useful addition to the main text. The link between the absolute numbers presented here and the percentage-based results in the text is still rather opaque however, and could be much more clearly signposted, both by including absolute numbers underlying all %s reported in the text and including the % data in Table 3. For example, I believe it is possible to derive the reported sensitivities of the two algorithms (line 160), e.g., for analyze high or medium risk classification/total malignant/pre-malignant is (212+239)/473 = 95.35%, but it does not seem to be possible to similarly derive the numbers for the reported specificities. Similarly, the data underlying the reported accuracies (76% for detect and 72% for analyze) ‘for malignant lesions compared to benign lesions’ cannot be derived. I believe that readers should not have to do so much work to try to understand the authors data and much more could be done to make it more accessible. Reporting of accuracy alone also makes it impossible to understand the source of and importance of the misclassifications – i.e., whether they are due to false negative or false positive results on the classifier. One does not want to risk missing malignant lesions however the potential health service implications from large numbers of false positive results cannot be under-estimated and must be taken into account.

**Author response: We thank the reviewer for these comments.**

**As you suggested we have included the absolute numbers in the text and the underlying %s in the tables 3a-c (see lines 133-136 and updated tables 3a-c).**

**Thank you for your concern about not to miss malignant lesions and the possible impact of a large number of false positive results on the health systems. Regarding that point, please refer to the study done by Tran et al. from 2005, who investigated the diagnostic accuracy of 272 general practitioners (GP) and six dermatologists in various skin conditions. Following their data, the overall diagnostic accuracy of the GPs was much lower than that of our investigated algorithms.**

**Of course, no neural network will achieve a diagnostic accuracy of 100%, however, in comparison to abovementioned study, our neural networks surpassed the GPs; given that, our networks could have a positive impact on the health system as unnecessary visits and histological examination will be reduced. In this context, please also refer to a recent study done by Jinnai et al.**

**We have added that point to our discussion (see lines 230-238) and both studies to our references (number 47 and 48).**

3. As a final point, given the tertiary referral center nature of the study population, I have some concerns about overstating of the implications of this study for use of these algorithms outside of a hospital setting, i.e., by patients themselves, without suitable caveats regarding potential for harm.

**Author response: We thank the reviewer for this comment. You raise an important point as it is important to investigate, how valid such algorithms work outside a specialized hospital setting. For that reason, we have already performed a further study, in which exactly that issue (use of these algorithms in lay application) was examined. Preliminary results show that both sensitivity and specificity are lower as in this present study, however, are still above the reported ones of the general practitioners in the study mentioned at point 2 (see Tran et al. 2005).**

Reviewer #2:

Dear authors, thank you for the chance to review your article, which is improving a lot. You have addressed partly what I had in mind in the last review, mainly including a Table 3 with some needed information.
I still think that is important for the analyses between the 2 softwares that you developed to include a table (it may be Table 3d) that shows histopathology against risk groups determined by the 2 softwares. After all, the preferred analyses were the histopathology and we don't see the results comparing the risk assessment by the 2 softwares versus the histopathology results.
Regarding the comment on line 166, we don't see any figure or table showing the results of this difference between malignant and non-malignant lesions. It would be nice to see a confusion matrix comparing malignant x non-malignant lesions for both softwares.
Best regards

**Author response: We thank the reviewer for these comments.**

**According to your suggestion we have added tables 4a and 4b, which show histopathology against the risk groups for both algorithms.**

**Furthermore, we have created two crosstabulations (tables 3d and 3e) for both algorithms showing the algorithms´ risk * clinical category.**

Thank you for your help and re-consideration

Sincerely yours,

Teresa Kränke, MD (corresponding author)

Michael Tripolt, MD, MPH

**Conflicts of interest:** Michael Koppitz and Michael Tripolt share a company founded after finishing the study to produce a consumer-usable early skin cancer detection app. This does not alter our adherence to PLOS ONE policies on sharing data and materials. The remaining authors have no conflicts of interest to declare.

**Funding source:** The development of the algorithms was funded by the amiflow Ltd., Graz, Austria; however, the authors did not receive any salaries by this company nor did it have any influence on the study design, data collection and analysis, decision to publish, or preparation of the manuscript.
